# Rapid on-site nucleic acid testing: On-chip sample preparation, amplification, and detection, and their integration into all-in-one systems

**DOI:** 10.3389/fbioe.2023.1020430

**Published:** 2023-02-01

**Authors:** Jingwen Wang, Han Jiang, Leiming Pan, Xiuying Gu, Chaogeng Xiao, Pengpeng Liu, Yulong Tang, Jiehong Fang, Xiaoqian Li, Chenze Lu

**Affiliations:** ^1^ Key Laboratory of Specialty Agri-products Quality and Hazard Controlling Technology of Zhejiang Province, College of Life Sciences, China Jiliang University, Hangzhou, China; ^2^ Zhejiang Hongzheng Testing Co., Ltd., Ningbo, China; ^3^ Zhejiang Gongzheng Testing Center Co., Ltd., Hangzhou, China; ^4^ Institute of Food Science, Zhejiang Academy of Agricultural Science, Hangzhou, China; ^5^ Key Laboratory of Biosafety detection for Zhejiang Market Regulation, Zhejiang Fangyuan Testing Group LO.T, Hangzhou, China; ^6^ Hangzhou Tiannie Technology Co., Ltd., Hangzhou, China

**Keywords:** nucleic acid testing, rapid on-site detection, nucleic acid amplification, biosensor, microfluidic chip

## Abstract

As nucleic acid testing is playing a vital role in increasingly many research fields, the need for rapid on-site testing methods is also increasing. The test procedure often consists of three steps: Sample preparation, amplification, and detection. This review covers recent advances in on-chip methods for each of these three steps and explains the principles underlying related methods. The sample preparation process is further divided into cell lysis and nucleic acid purification, and methods for the integration of these two steps on a single chip are discussed. Under amplification, on-chip studies based on PCR and isothermal amplification are covered. Three isothermal amplification methods reported to have good resistance to PCR inhibitors are selected for discussion due to their potential for use in direct amplification. Chip designs and novel strategies employed to achieve rapid extraction/amplification with satisfactory efficiency are discussed. Four detection methods providing rapid responses (fluorescent, optical, and electrochemical detection methods, plus lateral flow assay) are evaluated for their potential in rapid on-site detection. In the final section, we discuss strategies to improve the speed of the entire procedure and to integrate all three steps onto a single chip; we also comment on recent advances, and on obstacles to reducing the cost of chip manufacture and achieving mass production. We conclude that future trends will focus on effective nucleic acid extraction *via* combined methods and direct amplification *via* isothermal methods.

## 1 Introduction

Nucleic acids are biomacromolecules formed of nucleotide monomers; they are among the most basic substances of life. Both deoxyribonucleic acid (DNA) and ribonucleic acid (RNA) play a vital role in the transmission of genetic information. The importance of nucleic acid detection is increasing in fields such as species authentication, point of care testing (POCT) (Choi et al., 2021), diagnostics (Zhao et al., 2021), food safety (Shang et al., 2020), and forensics (Han et al., 2015), among others. Compared with conventional immunoassay (Guo et al., 2020; Ma et al., 2020; Guo et al., 2021), nucleic acid testing could provide a lower detection limit after amplification, more accurate and convenient quantification, and higher specificity against pathogens that have similar shell structures and different genetic materials. In these applications, a very large number of samples need to be processed in limited time, which has prompted the development of many new detection methods. Even so, there remains a growing need for rapid on-site detection methods, since this approach provides the most reliable characterization of freshly collected samples and accelerates the entire testing procedure, with little dependence on costly apparatus. Moreover, rapid on-site methods are often easy to use and require less operator training, which means they also have advantages in civic applications. The ability to perform massive nucleic acid testing in limited time has attracted further attention due to the outbreak of COVID-19. Further development in the domain of rapid nucleic acid detection methods is crucial to improve the current health care system.

Rapid on-site nucleic acid tests generally consist of three steps: sample preparation, in which nucleic acid is extracted from the testing subject; amplification, in which the target nucleic acid is amplified *via* various methods; and detection, in which the amplified product is qualitatively or quantitatively analyzed. Most publications refer to “detection time” as the time required for the amplification and detection steps, neglecting sample preparation time. However, the three steps relate closely to one other and should not be discussed separately. For instance, the use of isothermal nucleic acid amplification methods may not dramatically decrease amplification time compared to traditional polymerase chain reaction, but it can greatly reduce sample preparation time.

Microfluidic methods have several benefits and have attracted extensive attention. They are widely used in sample preparation, amplification, and detection. On-chip methods enable automatic sample manipulation and require smaller amounts of reagent and less expensive testing devices. The high surface–volume ratio and small thermal mass involved in these methods also improve the efficiency of heat transfer during amplification. For these reasons, microfluidic methods are suitable for the development of novel rapid methods for on-site detection of nucleic acid. In recent years, an increasing number of “all-in-one” or “sample-in-answer-out” systems have been developed to perform each of these three steps on a single chip. Many review papers have been published on this topic. For instance, Wu et al. reviewed on-chip methods for DNA extraction, PCR amplification, and detection, but this was the most recent occasion on which on-chip methods were classified according to these three steps in a systematic review (Wu et al., 2014). Since then, vast improvement has occurred in isothermal amplification methods, which calls for an update. Other recent reviews have focused on different issues concerning related topics, such as on-chip cell lysis (Grigorov et al., 2021; Chen Y. et al., 2022; Petrou and Ladame, 2022), the role of magnetic beads in these steps (Chen et al., 2020a), on-chip PCR methods (Chen S. et al., 2022), the contribution of 3D printing to on-chip amplification (Tzivelekis et al., 2021) and detection methods for all-in-one systems (Li et al., 2021), and microfluidic sensors (Sharma and Sharma, 2022). Several other reviews have also focused on these steps without limiting their scope to on-chip methods; these have covered topics such as sample preparation (Jeon et al., 2018; Danaeifar, 2022), isothermal amplification (Obande and Singh, 2020; Pumford et al., 2020; Islam and Koirla, 2021; Maiti et al., 2022), and detection (Chiorcea-Paquim and Oliveira-Brett, 2021; Leonardo et al., 2021; Reyes et al., 2021; Bhat et al., 2022).

The aim of this paper is to offer a systematic introduction to on-chip methods of nucleic acid sample preparation, amplification, and detection that can be used in rapid on-site detection, and to discuss recent advances and challenges in the process of integrating these steps to develop an all-in-one system. The principles of various methods for cell lysis, nucleic acid purification, amplification, and detection are explained, with recent examples provided in each category. The examples selected either exhibit great potential in the domain of rapid on-site detection or employ novel designs that could help to improve performance, throughput, and integration or to reduce costs. In the final section, we discuss the integration of the three steps in all-in-one systems and present predictions on future trends for further development.

## 2 Rapid sample preparation methods

Samples containing target DNA or RNA fragments need to be pretreated prior to further processing. The protocol for pretreatment varies depending on nature of the sample (plant or animal cells, virus, bacteria, etc.), the environment of sample collection (tissue sample, saliva, blood, sweat, urine, etc.), and the subsequent test procedures that will be carried out. In general, however, it involves two processes: lysis (destruction of the plasma membrane) and purification (to separate the nucleic acid from inhibitors that can affect amplification). Below, we present recent advances in on-chip cell lysis and purification methods and discuss the key issues in combining these two processes on a single chip.

### 2.1 On-chip cell lysis methods

Plasma membranes can be deformed using a chemical reagent, mechanical force, electric field, or heat. Chemical lysis is the most common choice for on-chip lysis; under this approach, alkaline solution or surfactants are used to disrupt the plasma membrane. In alkaline lysis, the solution is first adjusted to produce an alkaline environment (often between 11.5 and 12.5 pH) so that hydroxide ions (OH^−^) can break down the fatty acid–glycerol ester bonds on the plasma membrane, causing it to become permeable. Subsequently, sodium dodecyl sulphate (SDS) is used to help dissolve the proteins and membrane. Although alkaline lysis is applicable to most cell types, its low reaction rate is a major disadvantage in rapid detection. Wang et al. selected *E. coli* and *E. durans* as models to study the efficiency of Gram-negative and Gram-positive bacteria lysis that could be achieved within 2 min (Wang et al., 2020c). They found that *E. durans* was barely lysed by homogeneous alkaline solution within such a limited period; *E. coli* was found to be lysed when pH reached 10, and was best lysed at a pH of 13. Further increase in the pH did not provide better results due to probable damage to the nucleic acid. In comparison, they reported better results with electrochemical lysis methods in 1 min. Surfactants or detergents, especially non-ionic surfactants that cause less damage to proteins and enzymes, are widely used for lysis of mammalian cells. However, surfactants need to be combined with lysozymes when used to lyse cells that have multiple outer layers, such as bacteria. The precise distribution and effective mixing of the sample and lysing buffer is the key issue in on-chip chemical lysis; Grigorov et al. (2021) have provided a detailed review of this question. Compared with a homogeneous liquid phase, water–oil droplets might provide better mixing and prevent loss of lysed content. For example, Shamloo and Hassani-Gangaraj, (2020) utilized a water–oil droplet-based chip and took advantage of secondary flows generated inside the droplet to provide better contact between the lysis buffer and sample and to keep the microchannel in good condition for reuse. Despite the convenience of chemical lysis, chips using this method often need to be equipped with a mixing unit to further decrease lysis time. Additionally, cell residues in lysis buffer might disrupt subsequent steps and have to be carefully removed.

Mechanical lysis physically destroys the plasma membrane *via* shear stress. In the bead milling or bead beating method, samples are ground with rigid beads made of glass, ceramic, metal, or metal oxides at a high speed; the efficiency of this method depends on the size, shape, and composition of beads (Claudel et al., 2021). Novel mechanical lysis methods combine nanoscale patterning with the use of acoustic or piezoelectric force to induce similar shear stress. Wang K et al. (2019) built an acoustofluidic device containing 180 pairs of sharp edges and completed cell lysis *via* application of high shear force created by an acoustic streaming effect. The surface of the channel was incubated with 5% Pluronic F-127 to prevent attachment of cell debris. Farooq et al. (2021) also used acoustic streaming to induce collision between nanowires for cell lysis. Lysis efficiency of 97% was achieved after 10 s of stimulation with power less than 1 W, which suggests that this could be a gentle and effective method of lysis. Nittala et al. (2022) fabricated silicon components with various micro-patterns (pyramids, pillars, ridges, dense pointed structures, and needles) and tested their lysis efficiency as driven by piezoelectric actuation. Zhang G. et al. (2021) developed a pillar array chip to trap and lyse red blood cells *via* acoustic wave. Simulations of streaming velocity near the pillars and shear stress around the micropillars were provided to help regulate cell lysis. The authors also noted that the pillar array was not able to effectively trap all the cells at high cell densities. In some studies, researchers have experimented with creating microbubbles inside channels instead of nanostructures, which could simplify the design and fabrication of microchips. Frictional force generated by the gas/liquid interface could create “cavitation microstreaming,” causing strong circulatory flow. Liu X et al. (2020) designed a microbubble array to lyse multiple cells with oscillating bubbles. The lysis efficiency of this system was measured at 97.62% after 1 min, similar to the results obtained by commercial chemical kits in 15 min. The primary drawbacks of mechanical lysis are the difficulty and high cost of fabricating delicate nanostructures and the decrease in lysis efficiency that occurs when cell debris clings to the surface of the microchannel.

Electrical cell lysis produces irreversible pores in the cell membranes through application of a high electric field. It has several advantages, in the form of short lysis time, simple channel structure, and low requirements for chemical reagents. Pandian et al. (2020) have laid out a method of predicting transmembrane potential at different coating thicknesses and voltages to help determine the optimal lysis parameters. Lysis efficiency of 98% can be achieved by following this guidance. More importantly, it is possible to selectively lyse cell membranes by controlling the strength of the electrical field, which enables the preservation of intracellular membrane structures such as the nuclear membrane (Jeon et al., 2018). Wu et al. (2020) created field gradients at the surface of polarizable active metallo-dielectric Janus particles with an external electric field, which led to selective electroporation of bacteria.

In thermal lysis, an external heater is used to continuously denature cell membranes. Qian et al. (2022) built a portable microfluidic system that lyses exosomes at 95°C for 10 min. Nguyen et al. (2022) applied a similar protocol for virus lysis. In addition, they used a vibrator to agitate the solution for 30s every minute during the heating process, which reduced the heating time from 15 min to 10 min. Bacterial cell walls are relatively more difficult to break down than those of mammalian cells, but this can still be achieved with a slight alteration to the process. Rizzo et al. (2021) applied vigorous agitation with magnetic beads that were used to capture bacteria and improved lysis efficiency. Unfortunately, the authors report only the optimal lysis parameters without indicating the extent to which agitation improved lysis efficiency. Given that the use of a heating process during nucleic acid amplification is unavoidable, thermal lysis can make direct use of the same heating device, which results in a greatly simplified system design. The greatest drawback reported for thermal lysis is the damage that it causes to certain proteins inside cells; although this may affect further analyses, it is not a major concern for nucleic acid detection. For these reasons, thermal lysis has good potential for use in rapid on-chip nucleic acid detection.

Due to the need for quicker and more effective methods of cell lysis, many studies have combined multiple lysis methods to produce further improvements. Thermal and chemical lysis methods could be combined with a simple mechanical method to facilitate heat transfer or provide better contact between the lysis buffer and sample.

### 2.2 On-chip nucleic acid purification methods

Purification methods can be classified into centrifugation, filtration, and magnetic methods. In addition, another direct approach is available, in which nucleic acid amplification is conducted without further purification. Centrifugation was one of the earliest purification methods to be used in biochemical research, and it lends itself perfectly to combination with a centrifugal microfluidic chip for nucleic acid purification. Homann et al. (2021) present a disk-like microfluidic chip for automatic preparation of *Mycobacterium tuberculosis* samples; conventional PCR tubes can be connected to the edge of the chip and purified samples collected for further examination. However, with the need for integration of additional steps on a centrifugal chip, channel and valve design become more complicated. Brassard et al. designed a test cartridge that combines pneumatic and centrifugal forces for fluidic control. This design results in a tenfold reduction in the amount of elution buffer used and a simplified channel pattern (Brassard et al., 2019). As a result, the costs of the reagent and chip fabrication are both reduced.

In filtration methods, a membrane is used to selectively filter the desired DNA sample from undesired lysate. A silica membrane can bind with DNA at a high salt concentration. Yoon et al. (2021) designed a syringe-based DNA extraction device that uses a silica membrane to separate out target DNA. In some cases, a commercially available filter is externally connected to the chip; this approach makes for an easy design process, but potentially presents problems with leaking (Ryzhkov et al., 2020). In other cases, the membrane serves as one layer of a sandwich structure. Lee et al. (2020) used nanoimprint technology to fabricate a silicon nitride filter membrane with a pore size of 200 nm. Under this type of approach, the membrane is tightly fixed using two sets of screws to form a sandwich structure with Teflon compartments and PDMS panels, and an electric field is used to drive negatively charged DNA and RNA across the membrane. The pore size of membrane is often in the μm range. If the microchannel is not properly sealed, the liquid tends to leak elsewhere before passing over the membrane. For this reason, on-chip filtration methods impose stringent requirements for microfabrication and assembly.

The magnetic method uses magnetic beads coated with silica or other probes that can form specific binding with target DNA; they also make use of magnetic force for further separation. Researchers have proposed various on-chip methods of this type to improve speed or efficiency. Xu et al. (2020) prepared three sub-channels for the sample, washing buffer, and eluate buffer. These sub-channels merged into a main channel, creating multi-laminar flow. By controlling the position of the magnetic field, magnetic beads carrying DNA can be made to pass through each flow sequentially. This work simplifies the procedure because the magnetic beads could pass through the main channel and provide effective separation within 3 s. Deraney et al. (2020) incorporated use of an electric field into an existing magnetic separation chip; DNA extraction yield was increased by 15% thanks to the electroosmotic flow. Finally, bio-specific molecular interactions, such as antigen–antibody interaction, can be used to specifically separate target DNA. Bi et al. (2020) combined magnetic beads with polyclonal antibody to detect *Salmonella* in processed duck meat products; capture efficiency was found to surpass 95%, and the enrichment process was completed within 20 min.

The direct amplification method is a technique that allows nucleic acid amplification to be carried out directly after extraction. It relieves researchers of implementing the purification process and reduces the loss of original template nucleic acids to a minimum. The key to direct amplification is to reduce the impact of inhibitors in the lysate. This can be accomplished by the development of inhibitor-resistant DNA polymerase-buffer systems or by diluting the lysate solution to lower the concentration of inhibitors. Chin et al. (2016) used Phusion *Pfu* as an inhibitor-resistant DNA polymerase in the detection of *Salmonella* in pork samples without purification. Hedman et al. (2009) tested several DNA polymerase-buffer systems as potential alternatives to the standard Ampli*Taq* Gold polymerase used in PCR, and identified three systems that offered better resistance against inhibitors in saliva samples. A later study by the same authors showed that a further increase in tolerance to inhibitors can be achieved by blending two DNA polymerase-buffer systems: Ex*Taq* Hot Start and PicoMaxx High Fidelity (Hedman et al., 2010). However, this approach increases the cost of the reagent and enzymes (by approximately 2–3 times) and is not always applicable for different types of target DNA. Dilution of the solution leads to a decrease in the concentration of both the target nucleic acid and inhibitors. Direct amplification is possible if the concentration of target nucleic acid remains above the detection limit while inhibitors are diluted to a safe level. Satya et al. (2013) extracted DNA fragments from leaf tissue and ramie stem tissue using NaOH and Tris/Tris–HCl/Tris–EDTA and used the results for direct PCR after dilution; their protocol enables sample preparation within approximately 10–12 min.

The extracted solution needs to be heavily diluted (about 1000 times) to reach the safe level of inhibitors for PCR. This requires a very large amount of target nucleic acid in the sample. Isothermal amplification methods are more tolerant of the presence of inhibitors, and the sample therefore does not require as much dilution (approximately 10 times is sufficient); or, in some cases, it may be possible to use the sample directly without dilution. Nguyen et al. (2022) demonstrated direct, on-chip RT-LAMP for the detection of SARS-CoV-2: a commercially available direct PCR kit was used to lyse the virus, and the lysate was directly transferred into a reaction chamber containing RT-LAMP reaction solution for isothermal amplification.

### 2.3 Integration of on-chip lysis and purification

**FIGURE 1:**
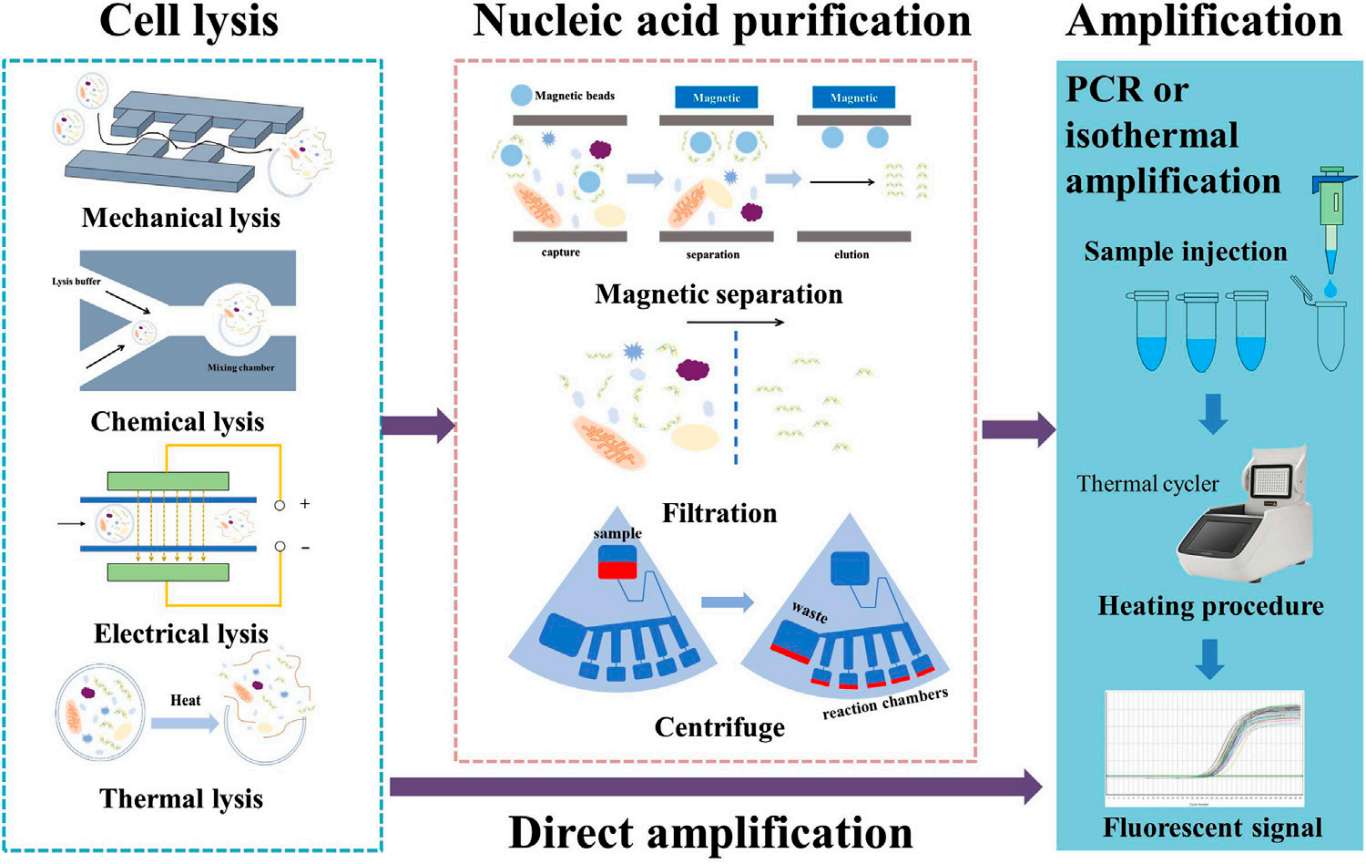
Various methods employed in nucleic acid sample preparation.

Figure 1 provides a general illustration of various methods of cell lysis and DNA purification. Chemical and thermal lysis methods do not need any special microstructures or chip accessories and are the most compatible with on-chip lysis, but they have limitations in terms of speed and efficiency of lysis. In order to resolve this issue, researchers have simply combined mechanical lysis with chemical or thermal lysis. Since mechanical methods only provide assistance under this approach, the fabrication of complicated microstructures can be avoided. Zhao et al. (2022) added a HeLa or HUVECs cell sample to a Chelex-100 suspension; the mixture was simultaneously heated and agitated. Agitation, as discussed above, can help to improve the effectiveness of thermal lysis. Furthermore, Chelex-100 particles were in full contact with the cells to perform mechanical lysis. The chitosan modification to these particles caused them to bond with nucleic acid immediately after lysis; they could then be used for purification. Kaba et al. (2021) made use of chemical lysis facilitated by a cavitation-microstreaming effect. They used a short-pulsed laser to create cavitation bubbles in the chemical lysis buffer, which simultaneously mixed the lysis buffer and crushed the cell membranes. Sun et al. (2021) added a chemical lysis buffer containing magnetic beads to a blood sample; gas bubbles were injected to fully mix the solution and to improve both chemical lysis and DNA binding.

Centrifugal purification is very readily adapted for use as part of a microfluidic chip driven by centrifugal force. As mentioned previously, channel design for a centrifugal chip becomes very complicated with the integration of more on-chip processes. A rotating chip is also not suitable for the application of an electric field. For these reasons, chemical or thermal lysis seem to be the only choices. On the other hand, its outstanding throughput is the main reason to select a centrifugal chip design. Magnetic purification methods not only offer high-selectivity DNA extraction, but also provide assistance in reagent mixing and fluidic control. This has become the most popular purification method for on-chip applications. However, the preparation of the magnetic beads and their coating layer is challenging and expensive. Chen et al. (2020a) present a thorough review of the preparation of magnetic beads for nucleic acid extraction.

Direct amplification methods require little pretreatment and can be combined with any lysis method. The current limitation with respect to these methods lies in the relatively high cost of commercial direct lysis or amplification kits for PCR. The high level of dilution involved in direct PCR also requires the presence of sufficient nucleic acid in the lysate. Due to their high tolerance to PCR inhibitors and outstanding speed of amplification, isothermal amplification methods have shown great potential for use in future studies. Current direct amplification methods mostly make use of commercial direct PCR kits, even in the case of isothermal amplification. We believe that more direct protocols for isothermal amplification will be developed in the coming years and that this will make direct amplification under isothermal conditions a feasible and economical solution for rapid on-site nucleic acid testing.

## 3 Rapid DNA amplification methods

### 3.1 On-chip PCR methods for rapid amplification

**FIGURE 2:**
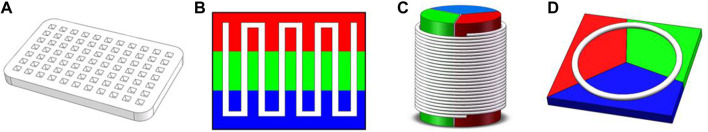
Common designs for PCR on a microfluidic chip: **(A)** stationary chamber chip; **(B)** serpentine channel chip; **(C)** spiral channel chip; **(D)** closed-loop chip. Colors represent different temperature zones (red: 95°C; green: 40°C–60°C; blue: 70–75°C).

Traditional PCR protocols consist of three steps: a denaturation step, in which the hydrogen bonding in the double-stranded DNA template is ruptured by exposure to a high temperature (95°C); an annealing step, in which the primer interacts with the template DNA at 40°C–60°C; and an extension step, in which the template DNA is amplified with the help of DNA polymerase at around 70°C. In some cases, the annealing and extension steps are merged to save time, but the entire process still takes approximately 1.5–2 h due to the repeated thermal cycles. Many portable microfluidic devices designed for PCR have been reported to reduce the required sample volume from 20 µL in traditional PCR protocols to 5–10 µL. The decrease in sample volume significantly decreases the thermal mass of the entire system. The high surface-to-volume ratio also enables more effective on-chip heat transfer. These features can speed up the process of applying the thermal cycles, reducing the time required from a few hours to approximately 30 min. Optimization of the heating/cooling process is crucial to the development of rapid amplification methods. Four common chip designs for PCR are illustrated in Figure 2; detailed accompanying descriptions are provided in this section.

#### 3.1.1 Stationary chamber PCR chips

Stationary chamber PCR is also referred as time-domain PCR: the solution is kept stationary while the temperature of the reaction chamber is modified to apply the necessary thermal cycles. Lee et al. (2005) developed a microfluidic chip that could complete 30 thermal cycles for a 15 μL sample within a total time of 26 min and 24 s. Taking a different approach, Muddu et al. (2011) heated the reaction chamber to different temperatures at each end and induced circulation of the reaction solution. The temperature gradient provided thermal cycling and enabled completion of the PCR reaction in 10 min. A stationary chamber PCR chip requires little space for a single reaction chamber; therefore, throughput of tests could be increased through integration of multiple reaction chambers on a single chip. Chips containing multiple reaction chambers are classified as multiple-chamber PCR chips. Matsubara et al. (2004) prepared a silicon-based array chip containing 1248 microchambers, with each chamber consuming only 40 nL of sample solution. Although multiple-chamber PCR chips provide high throughput, surface treatment of the inner walls of the chamber and a tight seal are often required to avoid cross-contamination. Moreover, microfluidic chips do not offer the same precision as complex PCR devices in terms of temperature control. Precision of temperature control has a strong impact on denaturation, enzyme activity, the efficiency of extension, and (most importantly) the specificity of amplification. Temperature gradient is more important for multiple-chamber chips, since an uneven distribution could also make comparison between different chambers untrustworthy.

#### 3.1.2 Closed-loop PCR chips

In closed-loop PCR chips, PCR solution is contained within a closed microchannel and periodically flows through three different temperature zones. The benefits of the closed-loop approach include easy control of the number of thermal cycles by simply changing the circulation time, a considerably shorter microchannel (which facilitates the construction of a miniature portable device), and good uniformity between each thermal cycle. Lok et al. (2012a) reported on the design of a magnetically actuated circular closed-loop PCR chip, in which a ferrofluid plug is driven by an external magnet that moves along the microchannel and four loops can carry out PCRs simultaneously. Espulgar et al. (2021) fabricated an identical closed-loop channel on a centrifugal disk-like chip; this design can apply 106 thermal cycles in 15 min. The drawbacks of closed-loop PCR chips are the inconvenience of retrieving the solution and an asymmetrical reaction procedure in concentric channels when high-throughput testing is attempted.

#### 3.1.3 Serpentine channel PCR chips

In serpentine channel PCR chips, tight bends are applied to a microchannel to form a series of connected parallel channels, while a heating module creates three temperature zones. Trinh et al. (2018) reported on the use of a serpentine channel on a PS surface with CNC milling; the surface of chip was able to capture bacteria with styrene groups, and *Escherichia coli* O 157:H7 was successfully detected in raw milk. The serpentine channel design is not limited to use with a traditional square shape; it can also be employed in the form of a radial circular chip. For instance, Zhang et al. (2022) designed a radial serpentine channel and heated it using a laser. The gradient of laser intensity created three temperature zones, in which a higher temperature was induced in regions closer to the center. The longer length of the peripheral part of the channel also provided a longer extension time. Additionally, the use of soft tubing instead of a manufactured microchannel enables alteration of the number of thermal cycles. Trinh and Lee, (2018) fabricated a micro-device with a glass–polytetrafluoroethylene (PTFE)–glass sandwich structure, in which a PTFE tube was twisted and fixed between two pieces of glass and acted as the microfluidic channel. This design enables greater flexibility in the number of thermal cycles.

Channel design has a direct impact on the time distribution of the denaturation, annealing, and extension steps. In order to increase extension time, Ragsdale et al. (2016) modified the width of the channels to impose a rapid transition from hot to cold regions and a slow transition from cold to hot regions. Li et al. (2020d) analyzed the influence of channel width-to-depth ratio and of the length ratio between the three temperature zones on temperature and flow distribution. Their results showed that the high temperature region should cover a larger area than the low temperature region to achieve optimal amplification of short DNA sequences.

#### 3.1.4 Spiral channel PCR chips

Spiral channel PCR chips use flexible tubes as a microchannel for the reaction solution. These tubes are twisted into a spiral shape and pass through multiple temperature sectors in a planar or cylindrical design. The number of cycles applied in each experiment is controlled by altering the length of the tube and the number of twists; hence, spiral channel PCR chips are more flexible than serpentine channel PCR chips. Wu and Wu, (2019) designed a portable PCR system based on a Teflon tube wrapped around a TEC chip; not only did the novel design reduce the size of the entire system, but the heat transfer rate was also 20%–30% faster than in conventional methods. Trinh et al. (2017) created a temperature gradient by positioning a PDMS mold swathed with PTFE tubing on a hot plate at constant temperature of 105°C; the temperature of the annealing and extension process was controlled *via* the height of PDMS mold.

Researchers have also used high-precision droplet manipulation techniques to create multiple droplets in the microchannel to increase throughput. Madadelahi et al. (2019) designed a high-throughput two-phase PCR device with a serpentine channel for droplet generation and a spiral channel positioned on a planar plate for thermal cycling; they concluded that such a device could reduce the time required for a PCR experiment by more than 40%.

### 3.2 Isothermal nucleic acid amplification chips

**FIGURE 3:**
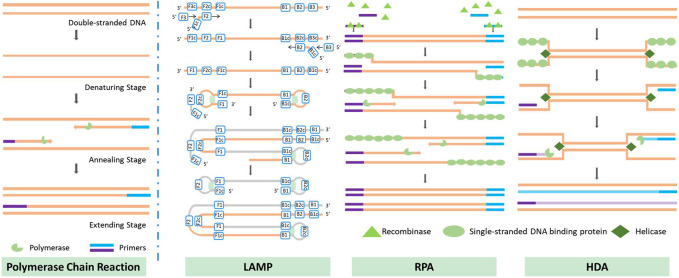
Principles of several methods of nucleic acid amplification.

Isothermal nucleic acid amplification methods have emerged during the last decade; these include loop-mediated isothermal amplification (LAMP), recombinase polymerase amplification (RPA), and helicase-dependent isothermal DNA amplification (HDA), among others (Pumford et al., 2020). Some isothermal amplification methods are not affected by PCR inhibitors, making them more suitable for rapid applications. Below, we present the principles underlying three isothermal nucleic acid amplification methods that are reported to have good resistance to PCR inhibitors (Kaneko et al., 2007; Kersting et al., 2014; Nixon et al., 2014; Wang et al., 2016), along with their on-chip applications, and discuss current challenges in on-chip amplification. The principles of the isothermal amplification methods discussed are also illustrated in Figure 3.

#### 3.2.1 Principles and comparison of isothermal amplification methods

##### 3.2.1.1 Loop-mediated isothermal amplification

Loop-mediated isothermal amplification was first introduced by Tsugunori et al. in 2000 (Tsugunori et al., 2000). The principle of the reaction is illustrated in Figure 3. LAMP uses six regions for initial recognition of the target sequence, and then uses four primers during the subsequent amplification, elongation, and recycling processes; this provides very high specificity. In some studies, two additional primers have been used to further increase specificity (Qin Y et al., 2021). Unlike PCR, which produces exact duplicates of the template DNA, the LAMP reaction produces dumbbell-shaped or stem-loop DNA structures with different lengths. Its product displays multiple stripes in electrophoresis tests. LAMP offers excellent efficiency in DNA amplification: Cornelissen et al. (2016) reported a detectable concentration of DNA after 8 min of amplification of a high-concentration sample (12 ng/assay) and after 60 min in the case of a low-concentration sample (1.2 fg/assay). Misir, (2018) compared the time required for amplification by 10^9^ to 10^10^ times *via* PCR and LAMP; the results showed that LAMP could complete this 1 h sooner than traditional PCR.

##### 3.2.1.2 Recombinase polymerase amplification

Recombinase polymerase amplification was first introduced by Piepenburg et al. (2006) in 2006. RPA begins with a binding process in which recombinase binds to a pair of primers, forming a nucleoprotein filament that can identify homologous sequences and create a D-loop structure to initiate a strand exchange reaction. Once the reaction is complete, the recombinase is released for the next pair of primers; eventually, two DNA duplexes are formed, and this process is repeated to amplify the target DNA. The recommended temperature for RPA is between 37°C and 42°C, which is the lowest temperature reported to date at which isothermal nucleic acid amplification has been achieved. The necessary temperature could be provided without the use of any heating device: in some cases, the temperature has been provided merely by body heat (Crannell et al., 2014), or the process has even been carried out at room temperature, with some sacrifice of accuracy (Shen et al., 2011). The amplification rate achieved by RPA is outstanding compared to other isothermal nucleic acid amplification methods. Xia et al. (2014) were able to amplify DNA to a detectable range within 10 min; in an efficiency comparison with the qPCR method, the same amplification result was achieved by qPCR after 134 min. Another method, known as “recombinase-assisted amplification” (RAA), makes use of a similar underlying principle to RPA; the main difference lies in the source of enzymes (Hou et al., 2022). In this review, we also include studies using RAA under the category of RPA.

##### 3.2.1.3 Helicase-dependent isothermal DNA amplification

Helicase-dependent isothermal DNA amplification was first introduced by Vincent et al. in 2004 (Vincent et al., 2004); HDA uses a DNA helicase enzyme, rather than high-temperature-triggered denaturation, to generate a single-stranded DNA template for primer hybridization, which enables isothermal amplification. Earlier work used an *E. coli* UvrD helicase/DNA polymerase I Klenow fragment pair with two accessory proteins (MutL and SSB) to amplify DNA at 37°C, and was referred to as mesophilic HDA (mHDA) for this reason. An et al. (2005) used a thermostable helicase, Tte-UvrD, together with Bst DNA polymerase to amplify DNA at 65°C; this version of the process was therefore referred to as thermophilic HDA (tHDA). Compared with mHDA, tHDA avoids reliance on accessory proteins and offers a more sensitive amplification process, making tHDA the more efficient method.

##### 3.2.1.4 Comparison of three isothermal amplification methods

Researchers always face the dilemma of how to choose from among the various isothermal amplification methods. Table 1 provides an overall comparison of the three isothermal amplification methods introduced above. In selecting the optimal method, the researcher must consider the length of the target sequence. Other than target length, the difficulty of primer design, amplification performance, and the cost of each test are also factors with a major impact on the use of rapid on-site detection methods. The cost is primarily dictated by the number and quantity of enzymes used during the reaction. Although on-chip methods may reduce the volume of reaction solution required, researchers have observed based on experience that there is a minimum amount required to successfully carry out the reaction. On the other hand, restrictions also arise from the detection method employed. For instance, LAMP-amplified products are fragments of different lengths with a repeating sequence of target nucleic acid; in this case, detection methods based on DNA hybridization probes cannot offer ideal detection. RPA-amplified products contain a large amount of enzyme residue, which will affect the result of electrophoresis or use of certain fluorescent dyes. For this reason, the detection of RPA-amplified products is often completed with the help of nucleic probes (Li et al., 2020b; Liu et al., 2021).

**TABLE 1:** Comparison of different isothermal amplification methods.

Amplification method	Reaction time	Preferable target length	Difficulty of primer design	Number of enzymes used	Reaction temperature
LAMP	20–60 min	<300 bps	Moderate	1	60°C–65°C
HDA	20–60 min	70–120 bps	Simple	2	37 or 65°C
RPA	10–20 min	<500 bps	Difficult	3	37°C–42°C

Although LAMP provides excellent specificity and an excellent amplification rate, its primary drawbacks are the limitation on target DNA length and the complex structure of the amplicon. The optimal target length for LAMP is less than 300 bps, and it is not recommended for amplification of target DNA longer than 500 bps (Pumford et al., 2020). As discussed above, the shape of the amplicon brings an added challenge to the detection process. However, the byproduct magnesium pyrophosphate also allows for indirect detection; this is discussed in the third section of this review.

The primary drawback of RPA is the high level of difficulty of designing primers. Since RPA operates at a relatively low temperature, secondary structures formed by longer primers are not denatured, and this could result in unsuccessful amplification; the use of primers within the range of 30–45 base pairs is recommended. Other key parameters affecting RPA primer design include GC content, target region, termini sequence, etc.; their influence have been discussed and explained in several articles (Daher et al., 2016; Lobato and O'Sullivan, 2018; Matthew et al., 2018). No report on automatic primer design software has yet been published, but the literature contains mention of several tools that could help researchers to screen several primer candidates for further optimization (Matthew et al., 2018; Li et al., 2020b). The second drawback of RPA is its limitation on the choice of target sequence. Although RPA is capable of amplifying long sequences, up to 1.5 kb, it is better suited to short sequences ranging from 80 to 400 bps, and preferably between 100 and 200 bps (Daher et al., 2016; Lobato and O'Sullivan, 2018). Fluorescent detection of RPA product is expensive due to the need for a fluorescence probe, which is why RPA product is often detected using lateral flow strip or other non-fluorescent detection methods.

**FIGURE 4:**
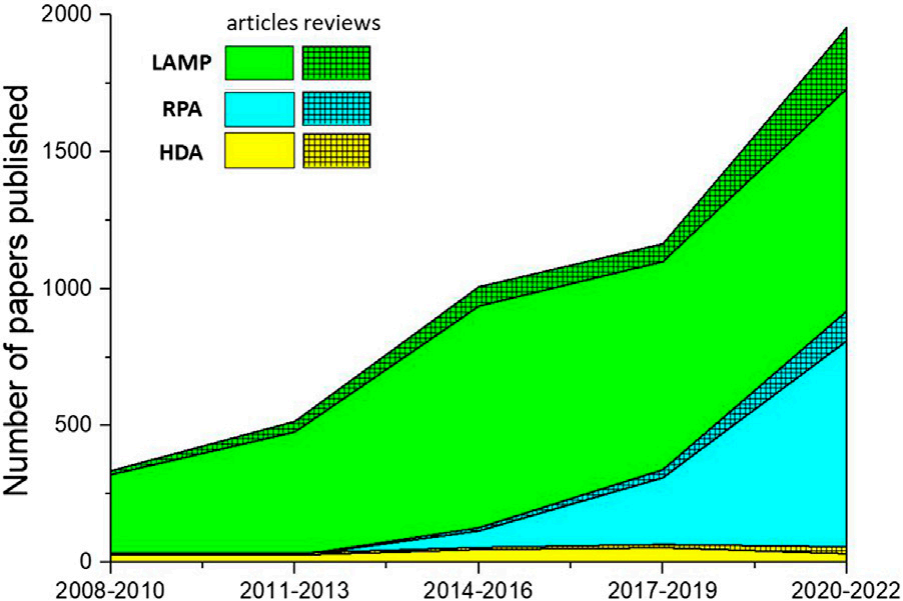
The number of papers on isothermal amplification methods published over the last 15 years. Solid color regions represent the number of research articles; hatched regions represent the number of reviews.

Figure 4 illustrates the number of papers published on the theme of various amplification methods; data were obtained from the core database of the Web of Science and the MEDLINE database on 7 November 2022. As this figure demonstrates, LAMP and RPA are drawing increasing amounts of attention, while HDA is declining in relative appeal. This is mostly due to obstacles in screening for more effective helicase. Because of the absence of a heating step, HDA is limited by the low denaturation efficiency of DNA helicase, leading to low specificity and an unstable amplification rate. Several studies have introduced nanoparticles in order to suppress primer dimer formation or to prevent binding between the template and proteins as a way to improve the specificity and speed of HDA (Chen et al., 2017; Sedighi et al., 2017). However, this greatly increases the cost of amplification, and researchers are still seeking more practical solutions.

#### 3.2.2 On-chip isothermal amplification

The reaction system for isothermal amplification is quite simple, which makes the corresponding chip design simpler than in the case of on-chip PCR. The same apparatus could be shared by most on-chip isothermal amplification methods, since the only difference in the amplification protocol is the heating temperature. The major difference in chip design arises from differences in sample preparation and detection strategy. Various pumping mechanisms have been used to mix the reagent and sample, such as centrifugal mechanisms (Soares et al., 2021; Zhou et al., 2021), a syringe pump (Hardinge et al., 2020; Natsuhara et al., 2020), a peristaltic pump (Najjar et al., 2022), magnetic force (Sharma et al., 2022), capillary forces (Zhu et al., 2020), etc. A serpentine channel is also used for amplification to provide homogeneous and effective heating. Tsougeni et al. (2019) carried out an HDA reaction in a 60 cm serpentine channel with a total volume of 30 µL. The results indicated that amplification efficiency was 96% relative to data obtained by using a conventional thermal cycler. In the case of direct amplification, several teams have introduced extracted sample directly into the reaction chamber without purification (Zhu et al., 2020; Nguyen et al., 2022). Novel fabrication methods have also proposed to ease manufacture of chips and reduce costs. Behrmann et al. (2020) used 3D printing to manufacture monolithic microfluidic chips for use in RPA reaction. They present an improved post-curing protocol to avoid autofluorescence and fluorescence drift, avoiding the need for further surface treatment.

Microfluidic methods are capable of achieving detection of multiple targets in the same sample by dividing the sample into several parallel reaction chambers (Yoo et al., 2020; Wen et al., 2022). However, the high amplification rate is likely to cause cross-contamination between each chamber. Most studies have used a thin film to seal the reaction chambers after sample injection (Wen et al., 2022), but this step also requires a skilled operator to avoid cross-contamination. Wax sealing could be used to seal each reaction chamber without requiring open-lid operation. Nguyen et al. (2022) installed wax valves (melting point 58°C–62°C) in each channel; after sample injection, a short period of heating was applied to melt the wax valves and seal the reaction chambers. Oil sealing does not require heating and is easier to control. Tian et al. (2022) preloaded mineral oil into syringes and injected this into each reaction chamber to complete the sealing process.

Many efforts have been made to increase test throughput. A disk-like centrifugal chip has a condensed fluidic pattern and can achieve very high throughput. Zhou et al. (2020) used a commercialized multi-channel disk chip containing four sets of microfluidic channels, each of which had eight reaction chambers. Their chip could test eight targets, including one negative control, in four different samples, and was used for rapid screening of emerging and re-emerging enteric coronaviruses in swine. Similarly, Nguyen et al. fabricated a centrifugal disk that could carry out 30 LAMP reactions for sex-typing (Van Nguyen and Seo, 2021).

### 3.3 Summary of on-chip methods of DNA amplification

The results of 40 studies of on-chip PCR systems are listed in Table 2. More studies than this were identified altogether, but we highlight only certain examples that are more recent or in which the system exhibited improved performance in rapid detection; the reader may find the remaining studies in the Supplementary Materials. Most of the PCR chips investigated were able to complete the reaction within 1 h, and the quickest could do so within 10 min. In general, continuous flow PCR chips are more time-efficient due to the removal of heating/cooling processes, and they can be easily connected to another microfluidic chip for detection of amplicon. Stationary chamber PCR chips have higher throughput. Based on the timelines reported in the listed studies, we draw the conclusion that the serpentine channel has become a mainstream design for on-chip PCR. The main reasons are the convenience of retrieving the amplification solution compared to a closed-loop structure and higher-precision temperature control compared with a spiral structure.

**TABLE 2:** Performance of 40 on-chip PCR systems. Total times marked with * indicate a value calculated based on protocols described in the relevant article; other values are quoted directly from the article.

Driving force	Number of cycles	Single cycle time	Total time	Limit of detection	Flow rate/Temperature change rate	Sample volume	Target	Potential for high-throughput testing	References
negative pressure	40	75 s	62 min*	—	—	—	Epidermal growth factor receptor gene mutation	NO	Yin et al. (2020b)
syringe pump	—	—	8 min 5 s*	—	0.1–2 μL/min	50 μL	Porphyromonas gingivalis Tannerella forsythia, Treponema denticola	YES	Yang et al. (2022)
liquid pump	40	90 s	72 min*	—	0.4 μL/min	5 μL	Colla corii asini	NO	Sheu et al. (2022)
syringe pump	27	120 s	56 min*	—	—	0.5 μL	Yeast expression vector	YES	Madadelahi et al. (2019)
syringe pump	30	—	—	—	0.773–0.889 mm/s	—	—	NO	Whulanza et al. (2017)
syringe pump	25	90 s	56 min*	≈ 10^2^ CFU	5 mL/min	10 μL	*Escherichia coli* O157:H7	NO	Trinh et al. (2018)
syringe pump	40	65 s	48 min 20 s*	10^2^ CFU/mL	0.25 mL/min	12 μL	Salmonella	NO	Wang Y et al. (2021)
—	40	60 s	<1 h	1 copies/μL	—	5 μL	Swine disease	YES	Jiang et al. (2021)
stepper motor	—	—	8 min	—	0.001–0.02 m/s	—	Bacterial 16S ribosomal DNA	NO	Li et al. (2020)
—	45	60 s	45 min*	50 copies	—	50 μL	HPV	NO	Zhu et al. (2019)
—	45	75 s	66 min 15 s	—	heating rate: 15°C/s cooling rate: 10°C/s	10 μL	HBV	YES	Battaglia et al. (2019)
syringe pump	25	15 s	<13 min	—	<0.5 mL/h	10 μL	Genetic markers	NO	Ragsdale et al. (2016)
syringe pump	50	26 s	—	—	—	10 μL	Human Genomic DNA template	—	Barman et al. (2018)
self-activated micropump	40	50 s	≈63 min*	—	50s/cycle	20 μL	H7N9	NO	Wang Z et al. (2019)
peristaltic pump	30	75 s	50 min	—	heating rates: 1.5°C/s cooling rates: −2.0°C/s	20 μL	HPV	NO	Liu et al. (2017)
stepper motor	35	40 s	13 min 20 s	125 CFU/μL	100 V/cm	50 μL	16S rDNA of periodontal pathogens	NO	Li et al. (2019)
peristaltic pump	30	10 s	<5 min	—	5 μL/min	25 μL	Mouse GAPDH housekeeping gene	NO	Moschou et al. (2014)
self-activated micropump	40	≈60 s	40 min*	—	8.5 μL/min	40 µL	HBV	NO	Wu and Wu, (2019)
syringe pump	40	60 s	<120 min*	10 copies	47 μL/min	0.1–10 μL	—	YES	Hatch et al. (2014)
hydrostatic pressure	30	—	—	—	0.006 mL/min	—	—	NO	Chen et al. (2016)
microfluidic pump	40	33 s	32 min*	10 copies/μL	—	70 μL	RNA virus	NO	Fernández-Carballo et al. (2018)
syringe/peristaltic pumps	35	85 s	85 min	—	heating rate: 6°C/s cooling rate: 4°C/s	6 μL	β-actin gene	YES	Cui et al. (2017)
syringe pump	27	120 s	50 min	—	—	10 μL	—	YES	Madadelahi et al. (2019)
syringe pump	25	29 s	≈15 min	—	—	25 μL	Synthetic pGEM-T vector inserted with the TTF-1 target gene	NO	Trinh et al. (2017)
syringe pump	30	—	<25 min	—	2 μL/min	20 μL	C. condimenti and *E. coli* O157:H7 DNA	NO	Trinh et al. (2016)
syringe pump	20	—	≈20 min	0.3 × 10^4^ CFU	2 μL/min	—	*E. coli* O157:H7 and Salmonella spp.	NO	Trinh and Lee, (2018)
peristaltic pump	36	—	≈30 min	—	1 μL/min	15 μL	Human cell lines: BC-3 and IBL-1	NO	Snodgrass et al. (2016)
syringe pump	25	—	—	—	≤1 mL/h	—	—	—	Thomas et al. (2017)
syringe pump	40	40 s	30–40 min	—	water phase: 0.4 mL/h oil phase: 1.2 mL/h	50 μL	HBV	NO	Li et al. (2020)
pneumatic pump	30	75 s	47.5 min*	—	—	10 μL	β-actin gene	YES	Trung et al. (2010)
magnetic force	25	36 s	20 min	1.63 copies/μL	—	0.5 μL	Bacteriophage lambda	YES	Lok et al. (2012b)
syringe pump	32	—	—	—	60 uL/min: 5 mm/s	—	—	NO	Ghalekohneh et al. (2020)
—	40	45 s	35.5 min*	—	20°C/s	100 nL	Ebola virus	NO	Ahrberg et al. (2016)
centrifugal force	35	150 s	89.5 min*	—	heating rate: 0.7–0.8°C/s cooling rate: 0.9–1.0°C/s	7.5 μL	Human serum	YES	Czilwik et al. (2015)
syringe pump	35	260 s	165 min*	—	400 μL/min	20 μL	C. albicans	NO	Fuchs et al. (2019)
piezoelectrically pumped	35	20 s	<15 min	5 fg/μL	45 μL/min	200 μL	*E. coli* DNA	YES	Haber et al. (2017)
motor	30	90 s	<30 min	—	—	5 μL	SSP150 DNA template	YES	Sugumar et al. (2012)
syringe pump	30	21 s	—	100 copies/mL	4 mm/s	55 μL	*Salmonella enterica Listeria* monocytogenes *Escherichia coli* O157:H7 *Staphylococcus aureus*	YES	Shu et al. (2014)
capillary force	50	—	≈30 min	—	—	50 μL	β-actin, *Escherichia coli* AH1pdm influenza virus Influenza virus H1N1	NO	Tachibana et al. (2015)
syringe pump	35	≈20 s	<30 min	—	0.8 mL/min	10 μL	KSHV/HHV-8	NO	Jiang et al. (2014)

On-chip PCR methods are facing two challenges. The first is their weak potential in the area of direct amplification. The second is how to manage the balance between simplicity of the device and high-precision temperature control. Efforts have been made to make sure that the temperature inside each reaction chamber is the same as the value measured by the sensor and to minimize the temperature gradient among different reaction chambers to improve uniformity of the results. In continuous flow PCR chips, the temperature gradient between different temperature zones may cause non-specific amplification. Several works have reported the addition of temperature gaps to isolate temperature zones, thereby reducing the amount of heat transferred. Other researchers have simplified the system by carrying out the extension and annealing steps in the same temperature zone (72°C). On the other hand, continuous flow PCR chips do not monitor temperature dynamically, which means a less complicated heating system is required. In addition, flow velocity has an impact on temperature control in continuous flow chips, because the solution carries heat to other temperature zones as it travels through the microchannel; thus, researchers should not unthinkingly increase the flow rate to reduce reaction time (Moschou et al., 2014).

Isothermal amplification methods enable nucleic acid amplification at a constant and moderate temperature (normally below 65°), and their specificity is not strongly affected by precision of temperature control. In some cases, sunlight and body heat can provide sufficient temperature control, which has facilitated the design of portable devices (Rathore et al., 2019). Moreover, on-chip isothermal amplification has displayed better potential for use with rapid detection than on-chip PCR methods. In Table 3, we list reported details of the performance of 30 on-chip isothermal amplification systems. In general, isothermal methods can save 10–15 min compared to PCR. Amplification time is generally between 15 and 40 min; in the quickest case, amplification can be completed in 5 min. Multiple reactions take place asynchronously without disturbance arising from denaturation. However, the high amplification rate is likely to cause aerosol contamination or carry-over contamination. On-chip methods can avoid open-lid operation, and they are considered to be a complementary strategy for isothermal amplification. Unfortunately, in most studies described in the listed articles, the amplicon was collected for further analysis, which would increase the possibility of false positive or aerosol contamination. Moreover, the three isothermal methods discussed here are highly tolerant to the presence of inhibitors in the lysate, which makes them suitable for direct amplification and could further reduce the total time to detection.

**TABLE 3:** Performance of 30 on-chip isothermal amplification systems.

Amplification method	Driving force	Limit of detection	Dynamic range	Amplification time	Detection method	Sample volume	Target	References
LAMP	centrifugal force	0.5 copies/μL	0.5∼10^3^ copies/μL	60 min	fluorescence signals	4 μL	SARS-CoV-2	Tian et al. (2020)
LAMP	capillary force	100 fg/μL	1 ng/μL∼100 fg/μL 10^8^∼10^2^ CFU/mL	45 min	gel electrophoresis	10 μL	Cryptococcus	Tian et al. (2022)
LAMP	syringe pump	MYSV: 11.1 ng/μL CCYV: 9.6 ng/μL	—	20–60 min	fluorescence	3.1 µL	Tomato yellow leaf curl virus, melon yellow spot virus, cucurbit chlorotic yellows virus	Natsuhara et al. (2020)
LAMP	centrifugal force	*E. coli*: 0.0134 ng/μL Salmonella spp.: 12 CFU/mL	—	30 min	gel electrophoresis	100 μL	*E. coli*, Salmonella spp., *Staphylococcus aureus*, Vibrio parahaemolyticus	Zhang M et al. (2021)
LAMP	centrifugal force	10^3^ copies/μL	10^3^ ∼ 10^6^ copies/μL	30 min	fluorescence	—	HPV	Zhao et al. (2021)
LAMP	syringe pump	10^1^ copies/μL	10^1^ ∼ 10^4^ copies/μL	45 min	fluorescence	—	HBV	Zhang et al. (2018)
LAMP	centrifugal force	100 copies/μL	—	within 45 min	colorimetric method	20 μL	Porcine epidemic diarrhea virus, transmissible gastroenteritis virus, porcine rotavirus, porcine circovirus type 2	Wen et al. (2022)
LAMP	—	50 ng/μL	—	40 min	fluorescence	14 μL	Carbapenemase-producing organisms	Wu et al. (2022a)
LAMP	—	10^2^ PFU/200 μL	10^1^ ∼ 10^5^ PFU/mL	40 min	fluorescence	2 µL	Dengue virus	Yoo et al. (2020)
LAMP	capillary force	—	—	30 min	colorimetric method	3 μL	SARS-CoV-2	Donia et al. (2022)
LAMP	capillary force	0.34 fg/μL	10–1 × 10^–4^ pg/μL	40 min	colorimetric method	1 μL	Prostate cancer 3 biomarker	Wang et al. (2020a)
LAMP	centrifugal force	10^2^∼10^3^ copies	10 ∼ 10^6^ copies/μL	30 min	fluorescence	10 μL	COVID-19	Soares et al. (2020)
LAMP	centrifugal force	10 copies/mL	10 ∼ 10^7^ copies/μL	40 min	fluorescence	—	SARS-CoV, MERS-CoV, SARS-CoV-2, HCoV-229E, HCoV-OC43, HCoV-NL63, HCoV-HKU1	Xiong et al. (2020)
LAMP	magnetic force	500 virions/mL	2.8 × 10^7^–28 copies/mL	45 min	colorimetric method	100 µL	HCV	Sharma et al. (2022)
LAMP	syringe pump	100 to 10000 copies/μL	5.5×10^4^–6.3×10^6^ copies/mL	within 30 min	fluorescence	6.4 μL	S. equi, S. zoo, EHV1 EHV4, EIV, H3N8	Sun et al. (2020)
LAMP	syringe pump	10^2^ per chamber	1.32 × 10^2^–.32 × 10^7^ copies	50 min	fluorescence	1 μL	*E. coli*, *S. Typhimurium*, V. parahaemolyticus	Nguyen et al. (2020)
LAMP	push pressure	14 CFU/mL	1.4×10^1^–1.4 × 10^6^ CFU/mL	30 min	turbidity of the reaction	250 μL	Viable Salmonella	Wang et al. (2020b)
RPA	capillary force	1 copy/μL	—	15 min	lateral flow detection	30 μL	COVID-19	Liu et al. (2021)
RPA	negative pressure	10 bacterial cells	—	30 min	fluorescence	10 μL	*E. coli*, *Listeria* monocytogenes, *Salmonella enterica*	Yin et al. (2020a)
RPA	centrifugal force	1 copy/μL	—	20 min	fluorescence	75 μL	SARS-CoV-2	Chen Z et al. (2022)
RPA	centrifugal force	S gene: 0.68 fM Orf1ab gene: 4.16 fM	—	10 min	fluorescence	1 μL	SARS-CoV-2	Cao et al. (2022)
RPA	centrifugal force	1.02 copies/μL	1.02–2.04 × 10^3^ copies	20 min	fluorescence	2.5 μL	Norovirus	Qin Z et al. (2021)
RPA	—	1 copy/reaction	—	5–10 min	fluorescence	5 μL	*L. monocytogenes*	Luo et al. (2022)
RPA	—	10 copies	—	15 min	fluorescence	—	HPV	Yin et al. (2020c)
RPA	syringe pump	—	—	10–30 min	gel electrophoresis	1 μL	*E. coli*	Georgoutsou-Spyridonos et al. (2021)
HDA	push pressure	10 CFU	10^5^ ∼ 10^1^ CFU	30 min	fluorescence	50 ng DNA	*E. coli*	Mahalanabis et al. (2010)
HDA	—	100 CFU	3∼more than 100,000 CFU/swab	60 min	chip image	5 uL	*Staphylococcus aureus*	Frech et al. (2012)
HDA	—	1 CFU	1–250 CFU/reaction	45 min	colorimetric method	2 μL	mecA gene	Pasko et al. (2012)
HDA	—	1.25 × 10^−2^ pg	125–2.5 × 10^−3^ pg	30 min	gel electrophoresis	5 mL	*Clostridium difficile*	Huang et al. (2013)
HDA	syringe pump	10 cells	below 500 cells	30 min	gel electrophoresis	100 μL	Foodborne pathogen	Tsougeni et al. (2019)

For both amplification methods, the primary obstacle for testing of multiple targets is the addition of different primers to multiple reaction chambers. In some studies, primers have been freeze-dried and preloaded to avoid cross-contamination (Chen et al., 2020b; Nguyen et al., 2022). In continuous flow PCR chips, the reaction solution could be separated with an oil phase in a similar way to the method used in digital PCR to test multiple targets (Jia et al., 2018). However, the surface tension needs to be carefully calculated and high-precision flow manipulation is required to keep the droplets in shape.

## 4 Rapid on-chip detection methods

In this section, we discuss the principle and application of several rapid on-chip methods for the detection of different types of amplicon. The performance of these methods is evaluated according to their speed, detection limit, equipment requirements, operational difficulties, and potential for mass production and high-throughput testing.

### 4.1 Lateral flow assay

Lateral flow assay (LFA) or lateral flow detection (LFD) has been widely used in the analysis of DNA amplicon; it could be regarded as the simplest paper-based microfluidic detection method. The most common type of lateral flow assay comes in the form of test strips that contain a sample pad, conjugate pad, nitrocellulose filter membrane with two test lines (for the sample and a positive control), and absorbent pad. First, the amplified DNA sample is injected onto the sample pad; the strip is then put into the migration buffer. The buffer flows along the strip, driven by capillary force; the DNA sample conjugates with gold nanoparticles (AuNPs) or other labels to form a sandwich-like structure that is captured by recognition elements, such as antibodies or DNA probes, immobilized on the test lines. Finally, the redundant labels are captured on the control line to create a second red line that validates the result. Recent studies have optimized the detection limit of LFA strips to achieve a very low level. Yin et al. designed lateral flow assay methods for the detection of sheep- and pig-specific PCR products for purposes of meat authentication; the detection limit was reported to be 10 fg target DNA in each sample (Yin et al., 2016; Yin R. et al., 2020). The detection limit of LFA strips could be further improved by correlating the result with more sophisticated labels. For instance, Deng et al. (2018) used quantum dots in place of AuNPs to enhance the sensitivity and accuracy of HIV-DNA detection; the detection limit reported was 0.76 p.m. Takalkar et al. (2017) used fluorescent carbon nanoparticles as labels on the DNA probe; the detection limit was 0.4 fM, 4-6 orders lower than achieved in LFA based on AuNPs. Another form of lateral flow assay makes use of a cotton thread-based format. Cotton thread-based devices have advantages in terms of their small size, low price, and ease of manipulation. The principle of cotton thread-based LFA is similar to that of strip tests. The strip is replaced by a cotton thread, while the nitrocellulose filter membrane is replaced by a layer of wax. Du et al. (2015) developed a cotton thread-based device for rapid detection of nucleic acid; the detection limit was reported to be 2.5 nM. Hydrophilic cotton thread modification could simplify the preparation of the sample pad and decrease the speed of lateral flow; such modification has been reported to produce a fourfold enhancement in the sensitivity of this method (Díaz-González and de la Escosura-Muñiz, 2021).

LFA is one of the most successfully commercialized detection methods, with a well-developed and cost-effective mass production technique and user-friendly protocol. LFA can be adapted for use with most nucleic acid amplification methods, including PCR, LAMP, RPA, and HDA, but it is most commonly used to analyze RPA amplicons. As mentioned above, fluorescent detection of RPA amplicons requires a fluorescence probe and is more expensive than other amplification methods (Bai et al., 2020). The result of LFA can be easily detected by the naked eye, and detection is not time-consuming. The detection times of four examples of LFA detection, listed in Table 4, are 2–5 min, 5 min, 10 min, and 5 min, respectively. For all these reasons, LFA strips have been widely used in qualitative testing (species authentication, clinical diagnosis, etc.) Recent advances in lateral flow detection have overcome its difficulties in multi-target detection and quantitative detection. Ma et al. (2020) used multiple-target lateral flow dipsticks to detect RPA-amplified *Staphylococcus aureus*, *Vibrio parahaemolyticus*, and *Salmonella* Enteritidis; the results were read using a handheld reader, and the detection limits were 2.6 × 10^1^ CFU/mL, 7.6 × 10^1^ CFU/mL, and 1.29 × 10^1^ CFU/mL, for each of these three targets, respectively. The total detection time, from amplification to reading, was 15 min.

**TABLE 4:** Performance of 30 on-chip nucleic acid detection systems.

Detection method	LoD	Linear range	Qualitative/Quantitative	End point/Real time	References
SERS	102.0 pg/L	—	qualitative	end point	Teixeira et al. (2020)
SERS	3–4 CFU/mL	1–10^8^ CFU/mL	quantitative	end point	Zhuang et al. (2022)
fluorescence	*E. coli*, *P. mirabilis*, and *S. typhimurium*: 1 CFU/μL *S. aureus*: 10 CFU/μL	—	quantitative	real time	Li et al. (2020c)
fluorescence	Singleplex assays: 2.5 × 10^1^ DNA copies for both khe and blaNDM-1 Duplex assay: 2.5 × 10^1^ DNA copies for khe and 2.5 × 10^2^ DNA copies for blaNDM-1	—	semi-quantitative	real time	Behrmann et al. (2020)
fluorescence	*Escherichia coli*: 17.15 ng/μL DNA *Staphylococcus aureus*: 5.67 ng/μL DNA *Pseudomonas aeruginosa*: 16.47 ng/μL DNA	*Escherichia coli*: 17.15–137.2 ng/μL DNA *Staphylococcus aureus*: 5.67–34.02 ng/μL DNA *Pseudomonas aeruginosa*: 16.47–197.58 ng/μL DNA	quantitative	end point	Guan et al. (2022)
fluorescence	1 × 10^1^ copies/μL	1 × 10^1^ to 1 × 10^4^ copies/μL	quantitative	real time	Zhang et al. (2020)
fluorescence	2.8 × 10 ^-5^ ng/μL	—	qualitative	end point	Zhou et al. (2022)
fluorescence	5 copies/μL	—	quantitative	end point	Yin et al. (2022)
fluorescence	89 CFU/mL	—	quantitative	real time	Wu et al. (2022b)
fluorescence	10 DNA copies	35 pg (10 haploid genome copies) to 350 ng (105 copies)	quantitative	real time	Khodakov et al. (2021)
fluorescence	1 × 10^1^ copies/μL	1 × 10^1^ to 1 × 10^5^ copies/μL	quantitative	end point	Meng et al. (2021)
fluorescence	10 bacterial cells	—	quantitative	end point	Yin et al. (2020a)
SPR	10 pg/mL	10^−5^ to 10^−12^ g/mL	qualitative	real time	Hsieh et al. (2022)
SPR	0.1 nM	—	quantitative	real time	An et al. (2021)
colorimetric	5 copies/μL	—	qualitative	end point	Dong et al. (2021)
colorimetric	—	—	qualitative	real time	Uddin et al. (2021)
colorimetric	—	—	quantitative	end point	Thio et al. (2022)
colorimetric	Sau and Sal: 10^2^ copies/μL; Sty, Pae, and Eco: 10^1^copies/μL	—	qualitative	real time	Liu D et al. (2020)
colorimetric	30 CFU mL^−1^	10^2^–10^7^ CFU⋅mL−1	semi-quantitative	end point	Yu Q et al. (2021)
colorimetric	100 copies/μL	—	qualitative	end point	Wen et al. (2022)
colorimetric	10^2^–10^3^ CFU mL^−1^	—	qualitative	end point	Jin et al. (2020)
quantum dot	0.11 pmol L^−1^	0.50 pmol L^−1^–50 nmol L^−1^	quantitative	end point	Kokkinos et al. (2018)
quantum dot	50 pmol	50–200 pmol	qualitative	end point	Nguyen et al. (2020b)
LFA	1 copy per μL or 30 copies per sample	—	qualitative	end point	Liu et al. (2021)
LFA	0.88 TCID50/mL	10^1^–10^3^ TCID50/mL	qualitative	end point	Chavan et al. (2019)
LFA	5 × 10^3^ CFU/mL	10^5^ CFU/mL to 10^3^ CFU/mL	qualitative	end point	Lu et al. (2020)
LFA	10–100 IU/mL	—	qualitative	real time	Bai et al. (2020)
electrochemical	10^2^ CFU/mL	10^2^ ∼ 10^6^ CFU/mL	quantitative	real time	Park et al. (2021)
electrochemical	7.4 fM	—	quantitative	real time	Liu et al. (2022)

### 4.2 Fluorescent and colorimetric detection

In the domain of nucleic acid detection, fluorescent dye can find applications based on its interaction with double-stranded DNA, or can be incorporated alongside other nanostructures as a fluorescent label. Fluorescent dye can directly emit light distinguishable by the naked eye, thus providing visual confirmation of DNA amplification, but the light signal increases greatly under excitation by UV light. Many sensitive devices, such as a CCD camera, fluorescent microscope, or UV-Vis spectrometer, are used for thorough analysis of fluorescent signals, but these devices often require a stable workplace to prevent interference from external light or oscillation. As a result, there are two approaches used in fluorescent rapid detection methods: qualitative end-point detection with visual confirmation; and quantitative or semi-quantitative real-time detection with portable devices.

Zhou et al. (2022) prepared a multi-chamber chip with a paper-disk and thin PDMS film for simultaneous detection of *Escherichia coli* O 157:H7, *Salmonella* spp., and *Staphylococcus aureus*. A portable device based on a smartphone was fabricated for end-point detection. Two UV lights powered by the smartphone battery were installed in a 3D-printed dark box; parallel installation meant that they provided uniform excitation. The detection signal was collected and analyzed by smartphone. The entire detection set cost only $120 and weighed less than 300 g; the detection limit was 2.8 × 10^−5^ ng/μL. Li et al. (2020c) used a high-intensity light-emitting diode (LED) to excite fluorescent dye and a photomultiplier tube to record the signal in real time. Eight reaction chambers carried out a LAMP reaction for four pathogenic bacteria and a negative control; the best LoD obtained for these targets was 1 CFU/μL. Fluorescent dye can also be used to detect byproducts of the LAMP reaction instead of binding diretly with amplicons. During the amplification process in LAMP, Mg^2+^ is consumed and turned into white precipitate. However, observation of this precipitate with the naked eye is not always reliable. In many studies, fluorescent dye (such as calcein or SYBR Green) has been added to create a more conspicuous light green color for easy detection. Zhang M. et al. (2021) embedded calcein-soaked paper in the reaction chamber for direct detection of LAMP products.

Digital nucleic acid detection uses an oil phase to divide the reaction solution into thousands of droplets prior to amplification. Each droplet contains either 0 or 1 original copies of the target DNA. As a result, the fluorescent signal obtained from these droplets can be processed using Poisson statistics to estimate the amount of nucleic acid in the original sample. This method provides rapid and highly accurate quantification of target DNA. Yin et al. (2020b) designed a self-priming digital PCR chip with a four-layer structure and six detection areas; this design enables the pre-introduction of specific reaction mix into certain detection areas and lowers the instrument and reagent requirements for multiplex detection. The use of digital methods is not limited to the domain of PCR; many on-chip detection methods have been reported for digital LAMP or digital RPA. Peng et al. (2020) designed a centrifugal droplet-based chip for application of digital LAMP. Traditional centrifugal chips suffer from droplet coalescence because the droplets are tightly packed. The design by Peng et al. optimized for droplet emulsification, using oil-storage structures to control the thickness of the oil film and prevent coalescence; the reported dynamic range was 10^1^ to 10^4^ copies per μL. However, digital methods require a long time to be spent on droplet formation and also require high-quality fluid control, which will affect the speed and cost of on-site detection. Luo et al. (2022) prepared a hydrogel nanofluidic chip for hydrogel RPA (gRPA) as a replacement for digital RPA. Under this approach, the movement of nucleic acids is restricted inside a hydrogel, and the end-point fluorescent image of the hydrogel can be used for quantification; this method reduces total time for amplification and detection to 5 min. A novel “random overlapping theory” was used instead of Poisson statistics to analyze the fluorescent image, and the detection limit was optimized to achieve detection of a single copy.

Despite its convenience, fluorescent dye is reported to slightly inhibit the PCR process and is more likely to bind with GC-rich sequences (Haukur et al., 2007). Moreover, fluorescent dyes are limited by their fluorescent efficiency, short lifetime, and risk of photobleaching. Quantum dots have been selected as an alternative to fluorescent dyes. Nguyen et al. (2020b) formed a quantum dot–DNA conjugate with semiconductor quantum dots and target oligonucleotides; this structure was used in a similar way to Forster resonance energy transfer (FRET) probes to quantify oligonucleic targets.

Fluorescent detection and colorimetric detection methods are capable of detecting multiple DNA targets through the careful design of labels that emit light signals at different wavelengths. Shen et al. (2022) fabricated a digital microfluidic device that detects multiple RNA targets at three different excitation wavelengths. This provides a visually clearer and more convenient method for multipurpose detection, but the high cost of multiple labels and high design complexity are disadvantages of this approach.

### 4.3 Non-fluorescent optical detection

Optical detection is based on various optical phenomena: emission, absorbance, and scattering of light, surface plasmon resonance, etc. The most intriguing advantage of optical detection methods is their high precision in the implementation of quantitative analysis. On the other hand, optical detection is highly demanding in terms of the need for a stable and lightproof platform to avoid disturbance from the working environment. Although the components required to control the optical behavior of the system and maintain a suitable working environment are rather expensive, some studies have succeeded in applying these methods in rapid on-site detection.

Surface-enhanced Raman spectroscopy (SERS) involves enhanced Raman scattering of molecules absorbed on or near a SERS-active surface; it is widely used in sensitive detection of biomolecules. Zhuang et al. (2022) designed a microfluidic paper-based device (μPAD) for super-sensitive SERS detection of RPA amplicons. Under this approach, SERS probes were first introduced to the μPAD; a Cas12a trans-cleavage system was added after drying. The solution then migrated within the μPAD and reached the detection zone; finally, SERS was used for sensitive detection. The entire process took less than 15 min. Teixeira et al. (2020) developed a microdroplet-LAMP-SERS platform for real-time detection of foodborne pathogens. The various lengths of LAMP products lead to variability in their SERS fingerprints, which makes direct detection very difficult. Multifunctional gold nanoparticles were used as a Raman reporter and Mg^2+^ chelating agent, enabling indirect detection of insoluble LAMP byproducts.

Surface plasmon resonance (SPR) is a phenomenon caused by the evanescent wave formed when monochromatic light reflects off the surface of a thin metal film (Lu et al., 2021). An SPR sensor measures the refractive index, angle of refraction, or energy of the reflected light to deduce mass change on the thin metal film. Its signal is highly proportional to the mass attached to the metal surface and provides highly accurate quantification of this. Hsieh et al. (2022) combined microfluidic PCR with the use of an SPR sensor to detect DNA sequences. DNA probes were immobilized on an SPR chip to capture PCR-amplified target sequences; the detection limit was as low as 10^−11^ g/mL. The signal obtained within 36 min was equal to the signal obtained after 105 min using traditional methods, representing greatly improved sensitivity. An et al. (2021) designed a label-free multi-target regenerable SPR sensor for DNA detection. Flow cells were first functionalized with dextran-streptavidin; subsequently, biotinylated probes targeting different sequences were immobilized on this layer, and the SPR chip could be regenerated after each use with NaOH and glycine-HCl. Their sensor is capable of being stably regenerated more than 100 times over the course of 20 days while maintaining good reproducibility of test results, which means that it could function as an economical long-term detection platform.

### 4.4 Electrochemical detection

Electrochemical detection of nucleic acid has a variety of advantages, such as rapid response, high selectivity, good portability, a relatively low cost, and a simple system design that comes with easy manipulation. Improvements in MEMS and nanotechnology have also brought about more options and better performance in the electronic components used to build electrochemical sensors and highly integrated devices for high-throughput testing. Under this method, electrodes are used to transduce biotic information into electrical signals, including impedance, resistance, voltage, and current.

Probes that can recognize a specific target are often immobilized on the surface of electrodes to generate or enhance the detection signal. Nucleic acid strands, antibodies, enzymes, and molecular imprint polymers are the most popular choices of probe. Methods of electrode surface functionalization have been well-developed in previous studies; the binding principles include but are not limited to gold–thiol self-assembly, biotin–streptavidin interaction, electrostatic adsorption, and amide bond formation (Baig et al., 2018). These probes are sometimes connected with other labels to further increase the system’s sensitivity and lower the limit of detection. The sequence-specific hybridization process of DNA is the most simple and effective probe design for detection of nucleic acid: under this approach, the complementary sequence of the target is functionalized on the electrode, and once the hybridization process begins, it will alter the electrical signal received by the sensor. Cinti et al. (2018) fabricated a paper-based strip for electrochemical detection of single- and double-stranded DNA, in which oligonucleotides tagged with methylene blue are immobilized on the surface to enhance charge transfer; the detection limit of this method is 3 nM for single-stranded DNA and 7 nM for double-stranded DNA. Instead of hybridizing the target sequence on the probe, researchers could form DNA structures without a target sequence, then break these and rebuild a more stable structure with the presence of target DNA in order to generate a signal. Zhang et al. (2018) immobilized two hairpin probes on the electrode surface, then opened the hairpin structures through hybridization of target DNA; subsequently, cyclic amplification of the DNA was triggered for signal amplification while the target DNA was released for the next cycle. This design enabled the simultaneous detection of two target DNA sequences at fM levels of concentration. The detection process could also target the byproducts of nucleic amplification, including electrons transferred during the process, in order to simplify the mechanism. Duarte-Guevara et al. (2016) designed a sensing technique that employs a field-effect transistor for transduction to detect foodborne bacterial pathogens after a LAMP process; rather than recognizing the amplified sequences, this method measures the pH change caused by the reaction.

The easiest way to implement on-chip electrochemical detection is to install electrodes at the bottom of the reaction chamber, or to dip the electrodes directly into the reaction solution. Park et al. (2021) designed an integrated circuit for use inside a pumpless microfluidic chip; PCR-amplified product was electrochemically detected *via* squarewave voltammetry. Screen-printed electrodes (SPEs) enable miniaturization of the electrodes; these are also low in cost and disposable, which is important in building a highly integrated on-chip detection system with highly repeatable results (Hernández-Rodríguez et al., 2020). Another possibility is to fabricate the circuit directly on the chip; this approach requires high-level microfabrication technology. For instance, Horny et al. deposited a Ti/Pt metal layer onto a glass substrate to function as electrodes, then deposited another layer of CNx for surface modification (Horny et al., 2020). This layer was combined with a PDMS layer to form a detection chip for microRNA.

### 4.5 Summary of on-chip DNA detection

In Tables 4 and Tables 5 we present a comparison of studies of on-chip DNA detection published in recent years and evaluate the general features of the detection systems examined. Novel developments in nucleic acid testing methods have focused on parameters including LoD, sensitivity, and linearity of the signal, among others. LoD is the most important of these parameters: a lower detection limit requires fewer cycles (or less time) for amplification, which improves the speed of detection. Optical detection methods offer the best precision, but they need to compensate for the errors arising from light passing through transparent chip material. Ideally, detection methods should be able to withstand external interference, such as from vibration or light, in order to achieve better on-site performance; optical methods require many precautionary measures in this matter. LFA is the method least affected by the working environment and offers a good LoD for qualitative detection (this can be as low as the pM range), but it is not suitable for quantitative detection due to the inaccuracies introduced by the use of handheld strip readers (Yu J et al., 2021).

**TABLE 5:** Overall evaluation of several detection methods.

Method	Characterization of quantitative accuracy	Throughput	Operator skill requirements	Equipment requirements	Cost per test
LFA	Qualitative/semi-quantitative	Low	Low	Low	Low
Fluorescent	Semi-quantitative/quantitative	High	Medium	Medium	High
Optical	Excellent	High	High	High	Medium
Electrochemical	High	Medium	Medium	Medium	Low

The introduction of an additional separation or cleavage procedure could be an effective way to improve the outcome of the detection process. For instance, on-chip detection methods sometimes add electrophoretic separation as a form of pretreatment to improve accuracy. Li et al. (2019) combined on-chip electrophoresis with a continuous flow PCR chip to achieve rapid separation and detection of pathogen genes. Due to the short migration distance, the total time required for on-chip electrophoresis is notably short. However, on-chip preparation of the necessary gel introduces extra steps, including gel formation, flushing, bubble removal, and sterilization, that may prolong the nucleic acid testing process. CRISPR-Cas has also been used in many on-chip detection studies. CRISPR-Cas can eliminate non-specific signals from amplification and is considered to be a complementary method for isothermal amplification. Chen Y. et al. (2022) used CRISPR-Cas12a-assisted RAA for SARS-CoV-2 detection. They introduced a short-strand reporter with a FAM fluorophore on 5′ and quencher on 3′. Every RAA amplicon could activate two CRISPR-Cas complexes; this increased the cleavage rate of the reporter sequence and released more fluorophore to enhance the signal. Similarly, Cao et al. (2022) used CRISPR-Cas13a to lower the LoD of RPA amplicons using LFA detection; the results were optimized to the fM level.

For on-site applications, any detection device is preferably highly integrated or simplified to minimize the overall complexity of the system. Electrochemical methods have strong potential to enable the fabrication of a highly integrated device, but research in this domain tends to focus solely on the detection step. The latest trend in electrochemical detection is the use of flexible materials to produce a wearable sensor for more convenient real-time monitoring (Mathew et al., 2021). This type of method is not compatible with on-chip sample preparation and amplification, because the direct fabrication of on-chip microelectrodes requires high-level MEMS techniques, and the implantation of flexible electrodes on a chip would mean the loss of its advantages in small size and flexibility. Handheld spectroscopic devices do offer advantages in terms of good portability, rapid response, and easy manipulation; however, minimization of the light source, probe, and spectrometer also leads to a decrease in precision. Increasingly many research groups have introduced smartphones into their methods of on-site detection, making full use of the camera for collection of data or images, the memory chip and hard drive for data processing, and the import and export interface for data transfer. Not only does this approach excel in terms of device size, it also provides a great advantage in terms of the ability to send data over WiFi or the Internet to achieve large-scale data processing.

## 5 All-in-one systems and future perspectives

With recent developments in on-chip methods of nucleic acid sample preparation, amplification, and detection, there has been an emergence of “all-in-one” or “sample-in-answer-out” systems that integrate all three steps into a single microfluidic system for automatic nucleic acid detection (Li et al., 2021). Below, we identify those studies that have aimed to develop methods of increasing the speed and accuracy of detection in this type of system and analyze their advantages and disadvantages; this is followed by a discussion of future trends and challenges.

### 5.1 Improving speed of detection

For most application scenarios, the priority of rapid on-site detection should first be to provide sufficient sensitivity and LoD to guarantee a reliable result; the next step should be to try to improve the speed and throughput of the system as much as possible. The three steps involved in rapid nucleic acid testing are closely related to one other, suggesting that simply concatenating the fastest method for each individual step is not necessarily the optimal strategy. For instance, certain isothermal amplification methods have not been found to provide much improvement in reaction speed compared to traditional PCR methods; however, due to their high tolerance to inhibitors, these isothermal methods can save a great deal of time when they are combined with a direct amplification approach. Although many on-site applications do not require an extremely low detection limit, improved performance in the detection step could relieve the burden on the amplification step. In particular, if a lower LoD is achieved, fewer thermal cycles or less time will be required in the amplification step.

### 5.2 System integration

**FIGURE 5:**
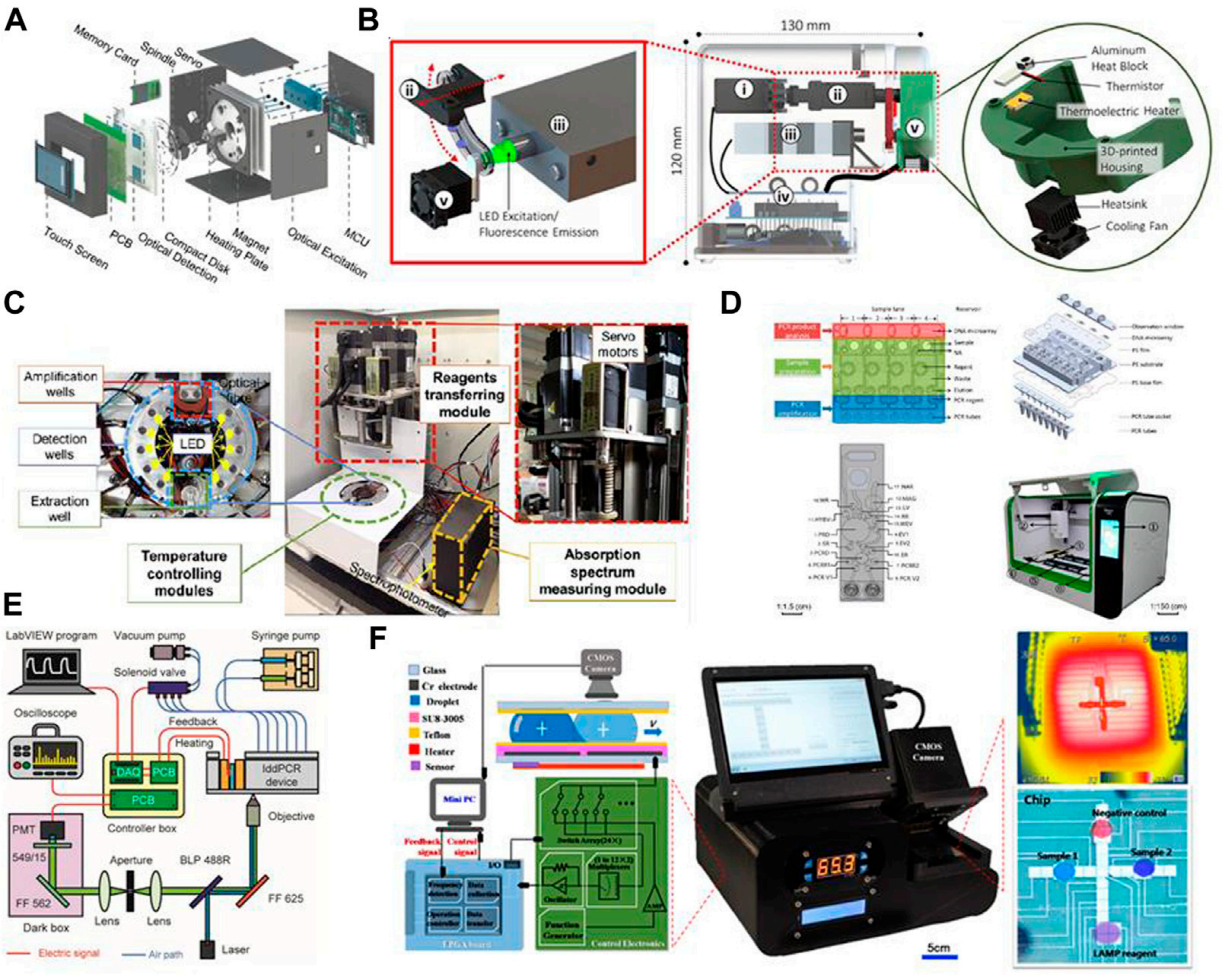
Graphical illustrations of several recently developed “all-in-one” systems and integration methods. Images are adapted from: **(A)** Choi et al., 2018; **(B)** Shin et al., 2018; **(C)** Dong et al., 2021; **(D)** Wang R et al., 2021; **(E)** Geng et al., 2020; and **(F)** Zheng et al., 2021. Permission to adapt and reproduce each image was obtained from the original publisher.

Some recent examples of system integration are presented in Figure 5 for a more vivid illustration of the possibilities. The easiest way to integrate the three steps is to connect separate chips *via* tubes. Tsougeni et al. (2019) connected three on-chip modules using PEEK capillary tubes; the cell lysis and DNA purification modules were fabricated on the same chip and connected to an amplification chip. This form of integration is easy to design, but a strong driving force is required to push the fluids through a long channel. Moreover, the diameter of the tube has to be very small (the aforementioned PEEK tube had an internal diameter of 150 μm and external diameter of 360 μm) due to the small volume of sample (in the range of 10 μL). A more common strategy is to design a multi-layer chip in which the three steps are completed individually in different layers (Li et al., 2020c; Tian et al., 2022). This design can help to reduce the size of chip *via* overlapping channels and can exploit gravity to avoid back-flow or cross-contamination.

Another issue is the pumping force through a long channel with tight bends. Although a single syringe pump may suffice, this imposes certain limitations in the case of a highly automated and multifunctional chip. Pumping mechanisms can also be combined to facilitate the entire procedure. Geng et al. (2020) used an electromagnetic valve-controlled peristaltic pump as well as a syringe pump; the former was used to transfer solution between different reaction chambers, and the latter was used to generate droplets for digital PCR. The drawback of combining two pumps is the large volume of the pumping system. Compared with a pneumatic peristaltic pump, which requires a vacuum pump, the choice of an electromagnetic valve-controlled peristaltic pump simplified the system design in this case. Centrifugal chips suffer from the same problem, since the centrifuge can only propel fluids from the center of the chip toward the edges. Malic et al. (2022) designed a centrifugal chip that was square-shaped rather than disk-like; a pneumatic pump was connected on one side of the chip to assist with fluidic control. Magnetic pumping is another solution to this problem, since it requires only a small piece of magnetic material and direct contact with the chip is not necessary. Furthermore, the magnetic beads involved in magnetic pumping could also help with mechanical lysis or DNA separation in the previous steps, which would make the entire system more effective.

A heating device is necessary for both PCR and isothermal amplification. Many articles pertaining to on-chip PCR provide a simulation of the heat gradient to avoid non-specific amplification (Saeed et al., 2016; Zeng et al., 2016; Li et al., 2020d). Isothermal amplification is not affected by such issues, and this is another of its advantages over on-chip PCR methods.

For DNA detection, optical and fluorescent sensing methods require an extremely lightproof working environment. In most cases, a sealed box is provided to cover the chip or the entire system (Li et al., 2020c). Electrochemical detection involves either installing wires or inserting electrodes during preparation of the chip. Some researchers have constructed a chip around a set of microelectrodes to provide a reaction chamber, but as discussed in the previous section, this approach means losing the advantages of small size and flexibility. Another strategy is to use a printed circuit board (PCB) instead of microelectrodes for electrochemical detection; the size of a PCB matches that of a microfluidic chip, and it is much cheaper compared to the use of screen-printed electrodes (Nagaraj et al., 2014). In addition, although a paper-based chip is not suitable for sample preparation and amplification, an LFA strip could also be pre-planted inside the reaction chamber of a microfluidic chip for detection purposes (Liu et al., 2021). Many works have reported the introduction of smartphones to provide power, lamination, image recording, software control, data analysis, and data transmission. This approach could reduce the use of PCBs, thus reducing system size and allowing more time to be dedicated to hardware testing and development.

### 5.3 Concerns relating to cost-reduction and mass production

Questions about the cost of microfluidic methods never cease to create obstacles for their development, not only in the domain of chip manufacture but also in terms of the apparatus required for pumping and detection. Studies always claim that reducing the required amount of reagent could lower the cost, but the high cost of these methods mainly arises from the use of microchannels in the µm range and the new mask or mold required for any slight change during development. Methods such as photolithography are also expensive in terms of manufacture and maintenance of the working environment and apparatus. Thanks to recent advances in fabrication technology, an increasing number of researchers are making use of high-precision 3D printing or CNC milling to design and test prototypes (Trinh et al., 2018; Sanchez et al., 2022). Once the channel design is defined, mass production *via* injection molding (which allows for a finest possible structure of 10–100 μm) would reduce the price of manufacturing chips or cartridges. This could bring the cost as low as 0.1 to 0.5 dollars per chip. However, this price range may not seem likely to readers, for three reasons. First, this price is based on the authors’ experience in China, where we assume prices are cheaper than those of suppliers in other countries due to fierce competition. Second, it does not account for the materials and labor required for chip assembly, thin film sealing, surface treatment, sterilization, transportation, etc. Last but not least, most companies tend to increase their profit margins on microfluidic chips as a means of recovering the enormous costs incurred at the research stage and in preparation of the injection mold. However, in general, mass production of delicate microfluidic chips has become more feasible in recent years. It is important to point out that, even once a reliable channel design is established, a great deal of work will still be necessary in the die-sinking process (the process of preparing the injection mold), since this method requires a slope in most structures for better lift-out and any imperfection on the surface of the mold may lead to unwanted microfluidic turbulence.

Methods of chip assembly also play an important role in reducing the total cost. Traditional plasma bonding requires a highly skilled operator and assistance from a positioning device to achieve good accuracy in positioning. Furthermore, any dislocation in the assembly process may produce a defective product and is irreversible, which also increases manufacturing costs. In some studies, the chip has been assembled using screws or clips, which is reversible and user-friendly (Bhise et al., 2016; Satoh et al., 2018; Lee et al., 2020). Use of a multi-layer design also allows thorough and large-scale sterilization with ethylene oxide prior to assembly, which could further decrease preparation costs. In order to reduce the total cost of the chip, some researchers have installed lateral flow assay strips inside microfluidic chips for detection (Liu et al., 2021).

Based on the content of the above discussion, we believe that future trends in making speed improvements will involve the use of two lysis methods and direct amplification based on isothermal methods. Combined lysis may enable completion of lysis within 1–2 min with an efficiency greater than 90%, and direct amplification with isothermal methods may enable purification to be bypassed, without the process being affected by inhibitors in the solution. Regarding the detection step, the abovementioned methods have all been found to provide rapid (within 2 min) or even real-time responses, but further lowering their LoD could save on amplification time and increase the speed of the full procedure.
